# Changes over time in the effect of marital status on cancer survival

**DOI:** 10.1186/1471-2458-11-804

**Published:** 2011-10-14

**Authors:** Håkon Kravdal, Astri Syse

**Affiliations:** 1 Faculty of Medicine, University of Oslo, P.O. Box 1078 Blindern, 0316 Oslo, Norway; 2 Cancer Registry of Norway, P.O. Box 5313 Majorstua, 0304 Oslo, Norway

## Abstract

**Background:**

Rates of all-cause and cause-specific mortality are higher among unmarried than married individuals. Cancer survival is also poorer in the unmarried population. Recently, some studies have found that the excess all-cause mortality of the unmarried has increased over time, and the same pattern has been shown for some specific causes of death. The objective of this study was to investigate whether there has been a similar change over time in marital status differences in cancer survival.

**Methods:**

Discrete-time hazard regression models for cancer deaths among more than 440 000 women and men diagnosed with cancer 1970-2007 at age 30-89 were estimated, using register data encompassing the entire Norwegian population. More than 200 000 cancer deaths during over 2 million person-years of exposure were analyzed.

**Results:**

The excess mortality of the never-married compared to the married has increased steadily for men, in particular the elderly. Among elderly women, the excess mortality of the never-married compared to the married has increased, and there are indications of an increasing excess mortality of the widowed. The excess mortality of divorced men and women, however, has been stable.

**Conclusions:**

There is no obvious explanation for the increasing disadvantage among the never-married. It could be due to a relatively poorer general health at time of diagnosis, either because of a more protective effect of partnership in a society that may have become less cohesive or because of more positive selection into marriage. Alternatively, it could be related to increasing differentials with respect to treatment. Today's complex cancer therapy regimens may be more difficult for never-married to follow, and health care interventions directed and adapted more specifically to the broad subgroup of never-married patients might be warranted.

## Background

It is well known that all-cause mortality rates are higher among the unmarried, especially the never-married, than among the married [1]. A similar pattern is also found for cause-specific mortality [2-4]. In particular, unmarried individuals are overrepresented regarding violent deaths, and also have a considerable excess mortality from lifestyle-related disorders, such as cardiovascular disease.

During the last decade, a number of studies have shown that also prognosis following a cancer diagnosis is influenced by marital status [5,6]. This is presumably partly due to a poorer overall health at time of diagnosis in the unmarried population compared to the married. In addition, differences in treatments received and adherence to treatment regimens are likely of importance, with married individuals perhaps having a higher chance of satisfactorily carrying out a course of therapy compared to their unmarried counterparts [7]. Besides, married individuals appear more likely to present with earlier stages of tumors at time of diagnosis than the unmarried [8-10].

Some authors have also investigated the changes over time in the marital status differentials in all-cause or cause-specific mortality [11-16]. These studies have suggested an increase in the excess mortality among the unmarried, but the reasons for this development remain unclear.

The objective of our study is to find out whether there has been a similar strengthening of the association between marital status and cancer survival, as one might expect given the apparent deterioration in the health and use of health care among the unmarried relative to the married that is indicated by the differences in mortality trends. This issue has not received any attention in earlier studies. An increasing disadvantage for the unmarried with respect to cancer survival would be of concern in a supposedly egalitarian society with free public health available to all citizens.

We use register data that cover the last four decades and encompass the entire Norwegian population. In total, more than 440 000 men and women with 13 common cancer forms are included in our analyses. The data allow us to control for any marital status differentials in the stage of the tumor at the time of diagnosis. The remaining effects would thus be a result of the cancer patients' general health status at diagnosis or health behavior afterwards, or the treatments received. Because several authors have suggested that the association between marital status and all-cause or cause-specific mortality may vary across age [15,17-19], we have estimated some models separately for those who were diagnosed with cancer below age 70 and those diagnosed at a higher age (which were two almost equally large groups).

## Methods

All cancer cases in Norway have been registered by the Norwegian Cancer Registry from 1953 onwards [20]. Our study is restricted to the 441 556 women and men who were 30-89 years old when they were diagnosed with a first tumor of one of the following 13 forms between 1970 and 2007: stomach, colon, rectal, pancreatic, lung, breast (females only), cervical, uterine, ovarian, prostate, or bladder cancer, malignant melanoma, or central nervous system tumors. The latter group included also benign tumors, in accordance with common practice. Their inclusion had no impact on our estimates.

Data on marital status as of January 1 every year since 1970, date of death (if any), and dates of immigration and emigration (if any) were extracted from the Norwegian Population Register, complete from 1964 onwards, and linked to the cancer data by means of unique individual identification numbers after ethical review by the Norwegian Board of Medical Ethics. The highest educational attainment as of January 1 each year since 1980 was similarly added from the Education Register operated by Statistics Norway. Educational attainment prior to 1980 was extracted from the 1970 census. The cause of death was obtained from the Norwegian Cause-of-Death Register.

We estimated discrete-time hazard models, which is a common and convenient type of survival analysis. For each individual, a series of three-month observations was created, starting at the time of diagnosis and ending at the end of 2007 or when the person died, had lived ten years since diagnosis (an observation window commonly used when studying cancer survival), or emigrated, whichever came first. There is no need to split into even shorter intervals than three months; the same results were obtained with one-month intervals. Each observation included a number of variables that referred to the situation at the beginning of the three-month period, and the outcome variable was death from the cancer type under consideration within the three-month period (i.e. the so-called 'corrected survival' approach; see comments below). If the person died from another cause, the observations were censored at that time. Observations were excluded if the person did not live in Norway at the beginning of the period, and logistic models were estimated from the remaining observations (using the Proc Logistic in the SAS software version 9.2), separately for women and men. The statistical significance level was set at 5%.

A total of 113 906 deaths occurred within the 894 814 person-years of observation for men. The corresponding figures for women were 91 796 deaths within 1 166 316 person-years.

In a study of how marital status affects cancer survival one should control for tumor localization (here included as a categorical variable with 13 levels), because the unmarried may tend to develop other types of cancer than the married, with a better or poorer overall survival. In our study, for example, relatively many of the malignancies among the divorced are lung cancers, for which the prognosis is poor. All other covariates are also categorical. Five categories were defined for educational attainment: compulsory school (10 years), lower secondary education (11 years), upper secondary education (12-13 years), tertiary education up to and including the Bachelor level (14-17 years), and higher education (18+ years). Tumor stages were classified into four groups: localized, regional spread, distant spread, and unknown.

Marital status was defined as married, never-married, widowed, or divorced/separated, and referred to the beginning of the calendar year of the three-month observation. It is thus "current" marital status rather than marital status at the time of diagnosis, which has been considered in many other studies. The differences are not very large however: 10% of the currently divorced and 19% of the currently widowed were married at time of diagnosis, and only 1% of the currently married were not married at time of diagnosis. Using marital status at time of diagnosis rather than current marital status had no substantively important impact on the estimates.

Another factor was age at the beginning of the three-month interval, defined as age at the end of that calendar year. It was grouped into 5-year categories, running from 30-34 to 95-99 years. The calendar year of the three-month observation was grouped similarly: 1970-74, 1975-79, 1980-84, 1985-89, 1990-94, 1995-99, 2000-04 or 2005-07. Time since diagnosis was grouped into ten one-year intervals.

Because the intention was to analyze changes in the effects of marital status over time, we estimated models separately for five- or ten-year periods. In addition, an interaction term between period and marital status was included in some models estimated for all years 1970-2007 to see whether there was a significant linear trend in the effect of marital status. In these models, also the other covariates were interacted with period to ensure that a change in the effect of marital status did not merely reflect that the effect of some important covariate had changed.

## Results

Table 1 shows the estimates from a model for cancer patients of both sexes that includes marital status, age, year, education, cancer location, time since diagnosis, and stage. All categories of the unmarried have a significant excess mortality compared to the married.

**Table 1:** Effects (odds ratios (OR) with 95% confidence intervals (CI)) of socio-demographic factors and disease characteristics on cancer mortality among men and women diagnosed with 13 types of cancer at ages 30-89 in Norway 1970-2007, and number of deaths and exposure time in the various categories

Men	*OR*	***95% CI***	***Number of deaths***	*Number of three-month-observations*
CURRENT YEAR				
1970-74	1		8693	129750
1975-79	0.95	(0.92 - 0.98)	13518	267382
1980-84	0.83	(0.81 - 0.86)	15069	361980
1985-89	0.65	(0.63 - 0.67)	16357	422973
1990-94	0.56	(0.54 - 0.58)	16337	481531
1995-99	0.48	(0.47 - 0.50)	16377	561331
2000-04	0.38	(0.37 - 0.39)	15934	667958
2005-07	0.31	(0.30 - 0.32)	8901	472650
				
CURRENT AGE				
30-34 years	1		216	12246
35-39 years	1.30	(1.10 - 1.52)	574	31101
40-44 years	1.41	(1.22 - 1.64)	1038	49900
45-49 years	1.62	(1.40 - 1.87)	2159	78791
50-54 years	1.70	(1.48 - 1.96)	4060	131634
55-59 years	1.86	(1.61 - 2.13)	7114	221477
60-64 years	1.97	(1.71 - 2.26)	11090	347443
65-69 years	2.16	(1.88 - 2.48)	15956	485747
70-74 years	2.45	(2.13 - 2.81)	19752	623305
75-79 years	2.98	(2.60 - 3.42)	20990	631130
80-84 years	3.81	(3.32 - 4.38)	17169	469591
85-89 years	4.99	(4.34 - 5.74)	9650	229119
90-94 years	6.21	(5.35 - 7.21)	1375	42172
95-99 years	10.91	(8.01 - 14.85)	53	1899
				
CANCER SITE				
Stomach	1		12992	155748
Colon	0.37	(0.36 - 0.38)	11956	417431
Rectum	0.42	(0.41 - 0.43)	7729	272293
Pancreas	2.25	(2.18 - 2.32)	8366	41087
Lung	1.60	(1.57 - 1.64)	30937	254946
Prostate	0.28	(0.27 - 0.28)	25921	1370914
Bladder	0.32	(0.31 - 0.33)	6472	462371
Malignant melanoma	0.33	(0.32 - 0.35)	2899	270878
CNS	1.24	(1.19 - 1.29)	3812	110266
				
TUMOR STAGE				
Localized	1		34212	2058969
Regional spread	2.48	(2.43 - 2.52)	39512	663815
Distant spread	6.47	(6.34 - 6.60)	26351	219460
Unknown	2.00	(1.95 - 2.04)	11111	423311
				
MARITAL STATUS				
Married	1		75052	2443243
Never-married	1.27	(1.24 - 1.30)	11316	266821
Widowed	1.11	(1.09 - 1.13)	17379	438024
Divorced/separated	1.18	(1.15 - 1.21)	7439	217467
				
EDUCATIONAL LEVEL				
10 years	1		60443	1494813
11 years	0.93	(0.91 - 0.94)	27315	901854
12-13 years	0.91	(0.89 - 0.93)	11575	430191
14-17 years	0.87	(0.85 - 0.89)	8117	351366
18+ years	0.81	(0.78 - 0.83)	3736	187331
				
TIME SINCE DIAGNOSIS				
<1 year	1		43337	811212
1 year	1.77	(1.74 - 1.80)	31329	565314
2 years	1.22	(1.19 - 1.25)	13060	431705
3 years	0.95	(0.93 - 0.98)	7629	351824
4 years	0.79	(0.76 - 0.81)	5014	292190
5 years	0.67	(0.65 - 0.70)	3553	246294
6 years	0.60	(0.57 - 0.62)	2645	209977
7 years	0.52	(0.50 - 0.55)	1954	178209
8 years	0.46	(0.44 - 0.49)	1470	150986
9 years	0.45	(0.42 - 0.48)	1195	127844
				
**Women**	*OR*	*95% CI*	*Number of deaths*	*Number of three-month-observations*
				
CURRENT YEAR				
1970-74	1		6950	169823
1975-79	0.92	(0.89 - 0.95)	10897	388511
1980-84	0.81	(0.79 - 0.84)	12012	515077
1985-89	0.64	(0.62 - 0.66)	12878	576426
1990-94	0.53	(0.51 - 0.55)	12862	633453
1995-99	0.45	(0.44 - 0.46)	13153	718431
2000-04	0.36	(0.35 - 0.37)	13145	830083
2005-07	0.31	(0.30 - 0.32)	7629	555427
				
CURRENT AGE				
30-34 years	1		373	39701
35-39 years	1.16	(1.03 - 1.30)	1100	120196
40-44 years	1.21	(1.08 - 1.35)	2028	205603
45-49 years	1.23	(1.10 - 1.37)	3346	305468
50-54 years	1.33	(1.19 - 1.48)	5084	407423
55-59 years	1.49	(1.34 - 1.66)	7177	475825
60-64 years	1.61	(1.45 - 1.79)	9212	513062
65-69 years	1.71	(1.54 - 1.90)	10937	535223
70-74 years	1.95	(1.75 - 2.16)	13116	547429
75-79 years	2.24	(2.02 - 2.49)	14257	521012
80-84 years	2.74	(2.47 - 3.05)	12837	415136
85-89 years	3.56	(3.20 - 3.97)	8788	240845
90-94 years	3.83	(3.39 - 4.32)	1187	56263
95-99 years	7.93	(6.21 - 10.13)	84	3645
				
CANCER SITE				
Stomach	1		8406	107729
Colon	0.38	(0.37 - 0.40)	13681	514405
Rectum	0.43	(0.41 - 0.44)	5786	238757
Pancreas	2.15	(2.08 - 2.23)	7971	40748
Lung	1.51	(1.46 - 1.56)	12573	118929
Breast	0.23	(0.22 - 0.23)	17968	1698208
Cervix	0.29	(0.28 - 0.30)	3637	313690
Uterus	0.23	(0.22 - 0.24)	3125	381665
Ovaries	0.40	(0.39 - 0.42)	8996	318192
Bladder	0.51	(0.48 - 0.53)	2709	142568
Malignant melanoma	0.21	(0.20 - 0.22)	1845	350666
CNS	0.77	(0.74 - 0.81)	2829	161674
				
TUMOR STAGE				
Localized	1		21170	2642621
Regional spread	3.03	(2.97 - 3.08)	38148	1335383
Distant spread	9.42	(9.21 - 9.64)	23675	240187
Unknown	3.14	(3.05 - 3.24)	6533	169040
				
MARITAL STATUS				
Married	1		39640	2360415
Never-married	1.17	(1.15 - 1.20)	10258	437771
Widowed	1.06	(1.04 - 1.08)	32685	1185496
Divorced/separated	1.07	(1.04 - 1.10)	6943	403549
				
EDUCATIONAL LEVEL				
10 years	1		54133	2126969
11 years	0.92	(0.91 - 0.94)	24991	1381340
12-13 years	0.82	(0.79 - 0.84)	3752	309135
14-17 years	0.82	(0.79 - 0.84)	6070	509229
18+ years	0.76	(0.70 - 0.83)	580	60558
				
TIME SINCE DIAGNOSIS				
<1 year	1		31537	826957
1 year	1.75	(1.71 - 1.78)	25243	642934
2 years	1.20	(1.17 - 1.23)	11413	532725
3 years	0.92	(0.89 - 0.94)	6922	463826
4 years	0.71	(0.69 - 0.74)	4522	410720
5 years	0.58	(0.56 - 0.61)	3191	368019
6 years	0.49	(0.47 - 0.51)	2353	330757
7 years	0.44	(0.42 - 0.46)	1846	298355
8 years	0.38	(0.36 - 0.40)	1423	269196
9 years	0.33	(0.31 - 0.35)	1076	243742

**Figure 1:**
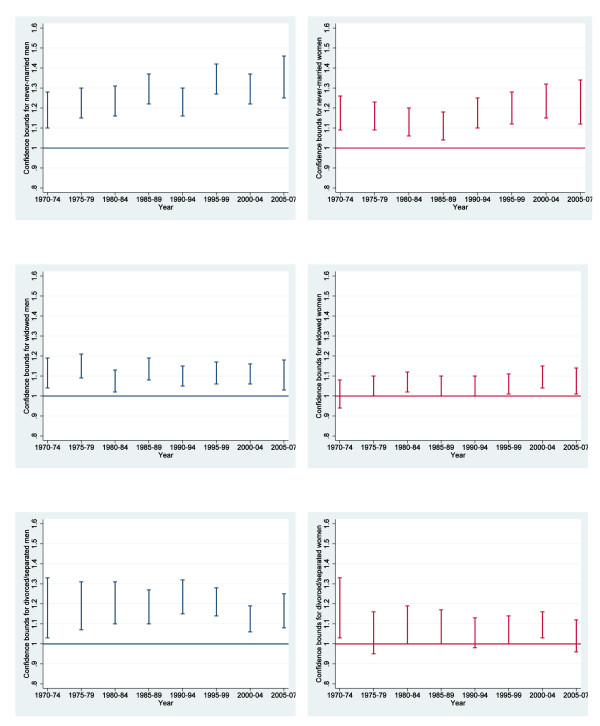
**Changes over time in 95% confidence bounds in excess mortality among unmarried (never-married, widowed, and divorced/separated) male (blue, left) and female (red, right) cancer patients compared to the married (reference line)**.

In Table 2 and Figure 1, we show how marital status affects mortality according to models that are estimated separately for men and women in five-year intervals (except the last one, which is for three years 2005-2007). For men, the excess mortality among the never-married relative to the married seems to have been increasing quite steadily over time. The situation for the divorced and the widowed, however, has not changed much. As for the women, the excess mortality across all categories of unmarried is roughly the same throughout the study period. The same patterns were observed when looking at ten-year rather than five-year periods (not shown).

**Table 2:** Effects (odds ratios (OR) with 95% confidence intervals (CI)) of marital status on cancer mortality among men and women diagnosed with 13 types of cancer at ages 30-89 in Norway 1970-2007, for different five-year periods^a^

Men	*OR*	*95% CI*
		
**1970-74**		
Married	1	
Never-married	1.18	(1.10 - 1.28)
Widowed	1.11	(1.04 - 1.19)
Divorced/separated	1.17	(1.03 - 1.33)
		
**1975-79**		
Married	1	
Never-married	1.23	(1.15 - 1.30)
Widowed	1.15	(1.09 - 1.21)
Divorced/separated	1.19	(1.07 - 1.31)
		
**1980-84**		
Married	1	
Never-married	1.23	(1.16 - 1.31)
Widowed	1.07	(1.02 - 1.13)
Divorced/separated	1.20	(1.10 - 1.31)
		
**1985-89**		
Married	1	
Never-married	1.29	(1.22 - 1.37)
Widowed	1.13	(1.08 - 1.19)
Divorced/separated	1.18	(1.10 - 1.27)
		
**1990-94**		
Married	1	
Never-married	1.23	(1.16 - 1.30)
Widowed	1.10	(1.05 - 1.15)
Divorced/separated	1.23	(1.15 - 1.32)
		
**1995-99**		
Married	1	
Never-married	1.34	(1.27 - 1.42)
Widowed	1.11	(1.06 - 1.17)
Divorced/separated	1.21	(1.14 - 1.28)
		
**2000-04**		
Married	1	
Never-married	1.29	(1.22 - 1.37)
Widowed	1.11	(1.06 - 1.16)
Divorced/separated	1.13	(1.06 - 1.19)
		
**2005-07**		
Married	1	
Never-married	1.35	(1.25 - 1.46)
Widowed	1.10	(1.03 - 1.18)
Divorced/separated	1.16	(1.08 - 1.25)
		
**Women**	*OR*	*95% CI*
		
**1970-74**		
Married	1	
Never-married	1.17	(1.09 - 1.26)
Widowed	1.01	(0.94 - 1.08)
Divorced/separated	1.17	(1.03 - 1.33)
		
**1975-79**		
Married	1	
Never-married	1.16	(1.09 - 1.23)
Widowed	1.05	(1.00 - 1.10)
Divorced/separated	1.05	(0.95 - 1.16)
		
**1980-84**		
Married	1	
Never-married	1.13	(1.06 - 1.20)
Widowed	1.07	(1.02 - 1.12)
Divorced/separated	1.09	(1.00 - 1.19)
		
**1985-89**		
Married	1	
Never-married	1.10	(1.04 - 1.18)
Widowed	1.05	(1.00 - 1.10)
Divorced/separated	1.08	(1.00 - 1.17)
		
**1990-94**		
Married	1	
Never-married	1.17	(1.10 - 1.25)
Widowed	1.05	(1.00 - 1.10)
Divorced/separated	1.05	(0.98 - 1.13)
		
**1995-99**		
Married	1	
Never-married	1.19	(1.12 - 1.28)
Widowed	1.06	(1.01 - 1.11)
Divorced/separated	1.07	(1.00 - 1.14)
		
**2000-04**		
Married	1	
Never-married	1.23	(1.15 - 1.32)
Widowed	1.09	(1.04 - 1.15)
Divorced/separated	1.09	(1.03 - 1.16)
		
**2005-07**		
Married	1	
Never-married	1.22	(1.12 - 1.34)
Widowed	1.07	(1.01 - 1.14)
Divorced/separated	1.03	(0.96 - 1.12)

^a^) The models also included current age, cancer site, tumor stage, educational level, and time since diagnosis.

We also conducted a trend analysis to explore possible changes in excess mortality, and this is portrayed in Table 3. For men, a statistically significant increase in excess mortality of about 3.4 percentage points per decade was observed for the never-married. No change could be detected for the divorced or widowed. For women, only rather weak indications of an increased excess mortality among the never-married and the widowed were seen. More specifically, a 1.8 percentage point increase per decade was seen for the never-married and a 1.4 percentage point increase for the widowed, but neither of these interaction effects were statistically significant. The corresponding interactions for the divorced were even further from being significant.

**Table 3:** Effects (odds ratios (OR) with 95% confidence intervals (CI)) of an interaction between marital status and period on cancer mortality among men and women diagnosed with 13 types of cancer at ages 30-89 in Norway 1970-2007^a^

**Men**	*OR*	*95% CI*	
Married	1		
Never-married	1.00341	(1.00136 - 1.00547)	p < 0.01*
Widowed	0.99976	(0.99795 - 1.00157)	p = 0.79
Divorced/separated	0.99892	(0.99631 - 1.00153)	p = 0.42
			
**Women**			
Married	1		
Never-married	1.00178	(0.99953 - 1.00404)	p = 0.12
Widowed	1.00137	(0.99966 - 1.00308)	p = 0.12
Divorced/separated	0.99853	(0.99584 - 1.00122)	p = 0.28

^a^) The models also included current age, cancer site, tumor stage, educational level, time since diagnosis, and the interaction terms between these variables and period.

Results from analyses stratified on age above or below 70 years at diagnosis are shown in Table 4. The increase in excess mortality for never-married men and women compared to the married is much higher for the older population. In fact, only the elderly display significant changes over time. Further, there are indications (p = 0.06) of an increasing excess mortality among the elderly widows compared to their married counterparts, while there has been a significant *reduction *in the corresponding excess mortality among widowed younger men. The excess mortality of others who are widowed or divorced has been stable.

**Table 4:** Effects (odds ratios (OR) with 95% confidence intervals (CI)) of an interaction between marital status and period on cancer mortality among men and women diagnosed with 13 types of cancer at ages 30-89 in Norway 1970-2007, according to models estimated separately for men and women diagnosed before and after age 70^a^

**Men**	*OR*	*95% CI*	
<70 YEARS			
Married	1		
Never-married	1.00210	(0.99914 - 1.00506)	p = 0.17
Widowed	0.99478	(0.99046 - 0.99913)	p = 0.02*
Divorced/separated	0.99861	(0.99532 - 1.00191)	p = 0.41
			
≥70 YEARS			
Married	1		
Never-married	1.00473	(1.00188 - 1.00760)	p < 0.01*
Widowed	1.00089	(0.99887 - 1.00291)	p = 0.38
Divorced/separated	0.99954	(0.99520 - 1.00388)	p = 0.84
			
**Women**			
<70 YEARS			
Married	1		
Never-married	1.00048	(0.99738 - 1.00358)	p = 0.76
Widowed	1.00088	(0.99806 - 1.00370)	p = 0.54
Divorced/separated	0.99812	(0.99481 - 1.00144)	p = 0.27
			
≥70 YEARS			
Married	1		
Never-married	1.00370	(1.00037 - 1.00704)	p = 0.03*
Widowed	1.00221	(0.99992 - 1.00451)	p = 0.06
Divorced/separated	1.00103	(0.99628 - 1.00578)	p = 0.67

^a^) The models also included current age, cancer site, tumor stage, educational level, time since diagnosis, and the interaction terms between these variables and period.

## Discussion

Our study shows that unmarried Norwegians with a cancer diagnosis have poorer survival (i.e. higher cancer mortality) than the married, in line with what has been reported previously [5,6]. The magnitude of this excess mortality has increased steadily for never-married men, in particular the elderly. A similar development is seen for older never-married women and elderly widows, while the excess mortality among the divorced, for both sexes, has been stable. Possible reasons underlying this development are discussed below, with attention first given to possible causal effects and then to potential selection mechanisms.

### Potential reasons for excess mortality among unmarried persons with cancer in general

It is possible that married individuals, because they are taken care of by their spouse, are more prone than the unmarried to visit a physician at occurrence of symptoms, thus possibly discovering tumors at an earlier stage [8-10]. Early detection may increase the chance of a successful treatment. It may, however, also be positively associated with measurements of survival simply by increasing the time between diagnosis and death (the so-called lead-time bias). As we control for such differentials, with some limitations discussed below, the remaining discussion is centered on other causal pathways.

One probable mechanism for the excess mortality among the unmarried is that they might have poorer overall physical health at time of diagnosis. In support of such a relationship, several studies have reported lower scores of self-rated physical health among the unmarried than the married [21,22]. An important reason for this pattern is probably that social support or pressure from the spouse and economic advantages achieved by sharing a household and having a spouse who contributes lead to a healthier lifestyle; with for example better nutrition and less smoking and alcohol abuse [1,23-25].

Also the mental health at time of diagnosis may affect cancer survival. Studies have shown that mental health problems are more common in the unmarried population, presumably in part because of lack of social and emotional support [1,24,26]. Common problems are e.g. depression, anxiety-disorders, and loneliness [26-28]. These conditions, perhaps the latter two in particular, may result in psychological stress [29]. This could in turn lead to more risky health behaviors and poor sleep, thus adversely affecting also the general physical health status [30]. Additionally, stress has been shown to have a more direct effect on physical health [4,31], and some studies even suggest effects on tumor growth [32], though there are also studies where such effects have not appeared [33].

In addition to physical and mental health, treatment is of course an important determinant of cancer survival. These factors are actually linked, because psychological stress and depression may cause poorer adherence to treatment regimens [30]. It is possible, even in a supposedly egalitarian country such as Norway, that married individuals receive better treatment from hospitals than the unmarried. Adherence to treatment regimens, however, is perhaps likely to play a more important role. A meta-analysis suggests that marriage influences adherence to treatment positively, partly through the partner's support [7]. Besides, one might expect that married individuals have a better chance of avoiding unhealthy behaviors after a diagnosis has been made, thereby improving prognosis [34].

In addition to affecting the survival prospects through factors such as spousal support, marriage may have an effect through parenthood. Raising children appears to have a positive effect on cancer survival [35], probably because children induce a healthier lifestyle and (especially if they are adults) may provide support during treatment and later. Unfortunately, our data only included information about children for the youngest individuals.

Finally, selection obviously contributes to the difference in cancer survival between married and unmarried individuals. For example, men with much knowledge and high income (potential) are seen as desirable partners and therefore tend to display high marriage rates (though not while studying) and low divorce rates, while the corresponding effects of women's socio-economic resources are more ambiguous and probably (as we return to below) have changed over time [36,37]. Education and income are also important determinants of health [38], and may through such differentials in health, or in treatment, also affect the cancer survival [39]. We have controlled only partially for this confounding effect of socio-economic resources by including education. Also, values may play a part. Individuals who are engaged in religious activities, for instance, appear more prone to avoid risky health behaviors [1]. In addition, they are less likely to divorce their spouses [40]. The values also include lifestyle preferences, with implication for entry into and out of marriage as well as health behavior. Next, healthy individuals are probably more likely to enter and remain in a marriage than the less healthy [41], although there are also studies indicating a negative health selection into marriage [23]. Furthermore, the health of the spouse is obviously a determinant of widowhood, and is linked to the health of the person under study. Finally, childbearing is not only a result of marriage; it is also a determinant. For example, married individuals with children, non-adult in particular, are less likely to divorce than those without [40]. As mentioned, children may affect cancer survival as well.

### Potential causes for increased excess mortality among unmarried cancer patients

When discussing trends in excess mortality, we first consider the never-married, for whom the changes have been most pronounced. In principle, changes in any of the mechanisms described above could help explain the observed increase in excess mortality in this group.

Starting with the health factors, it is possible that the never-married have had an increasingly poor health at the time of diagnosis compared to the married. In support of that idea, it has been shown by some researchers that the never-married have experienced a less favorable development in all-cause mortality over the last few decades [e.g. [11,13-16]] and in mortality from cardiovascular diseases [16,42], the latter in particular being indicative of growing differences with respect to health-related lifestyle. The very few studies that have investigated the changing differentials in self-rated health have provided mixed evidence. An American study found an especially pronounced health improvement in the never-married population compared to the married [43], while a Finnish study suggested the opposite [21].

The reasons for the relative deterioration in general health among the never-married are far from obvious. We can only offer some suggestions for why it may have become more important to have a spouse who provides support or exerts some pressure. One possibility is that the social cohesion in the society may have decreased over time [44]. A growing importance of self-realization in the population may have reduced the willingness to care for others aside family and friends [45], and increased workloads and work-related demands may have had similar effects. In a setting of reduced social cohesion, it is not unlikely that the never-married individuals would be particularly vulnerable. Especially the older never-married population might be at risk, considering that elderly individuals could have more difficulties in maintaining social connections outside the family than the younger.

The increasing excess mortality among the never-married cancer patients may in principle also be linked to the substantial improvements in diagnostic techniques. As mentioned, married individuals tend to be diagnosed with cancer at an early stage [8]. They are more likely to visit a physician at early symptoms of disease, and more eager to undergo examination even without feeling symptoms [e.g. [46,47]]. The latter has become an increasingly relevant issue because of the technological development, and the consequence may be that, among patients recorded with a localized tumor, the married have the smallest ones - those that to a lesser extent have infiltrated surrounding tissue. Although stage is adjusted for in this study, this control is not complete, as it does not account for sub-stages. It is, however, not likely that the possibly earlier detection of cancer among the married can contribute much in explaining the increasing excess mortality among the never-married. The estimates were very similar when we did not include tumor stage in the models (not shown), which suggests that additional control for sub-stages would also matter little.

To the extent that there are marital status differentials in treatment, it is not impossible that these have increased. One reason is that support from others may be important for a patient's compliance with the treatment recommendations, and that those without a spouse may find it increasingly difficult to find alternative sources of such support, as mentioned above. Considering that treatment regimens are more complex today than earlier, and that more care is performed in the outpatient setting, support in adhering to treatment is perhaps of particular importance nowadays. Furthermore, it seems to be a common perception among health personnel that their workload is increasing. If that is the case, it is not impossible that physicians perhaps are more likely to yield to pressure from next of kin, possibly giving married individuals an advantage in receiving better treatment.

Finally, there may have been a change regarding the selection factors. In particular, a number of studies have suggested that a high wage potential now increases a woman's chance of being married, while the opposite was the case a few decades ago, when specialization within the household (with the man having paid work and the woman taking responsibility for the housework) probably to a larger extent was considered a key advantage of marriage [37,48]. This change may not be adequately captured by the included education variable.

Regarding the widowed and divorced populations, trends in excess mortality are less clear than among the never-married. The arguments about partnership perhaps being more important for the general health because of weakened social cohesion in society - or even having a larger effect through treatment - should be relevant also for these groups. However, it is much more common among the divorced and widowed than among the never-married to have children, which may compensate for a lack of spouse. Furthermore, if there really has been a gradually more positive selection into marriage, leaving a less resourceful group of never-married, one would expect a similar increasing disadvantage among the divorced, because many of the factors that stimulate entry into marriage also tend to increase the chance of remaining in marriage [40]. The selection with respect to widowhood is very different. In marriages in general, a partner's health affects one's own health. A widowed person might thus have poorer cancer survival because of having shared an unhealthy environment with a spouse who deceased. It does not seem likely, however, that there has been much change in this mechanism during the time period in question.

### Methodological considerations

The major limitation in this study is that it has not been possible to distinguish between single and cohabitants within the unmarried population. Among elderly people, the proportion which cohabits is still low. For example, only 3% cohabited at age 70-79 in 2010, while one-third were unmarried [49]. The increasing prevalence of cohabitation among the younger in our study population, however, has implications for the interpretation of the estimates. For example, 13% were cohabitants at age 50-54 in 2010 - after a doubling over the preceding 15 years - in comparison with 40% unmarried [49,50]. (The corresponding figures at age 30-34 were 30% and 62%, but this is less relevant for our analysis because of the few cancer cases in that age group.) If cohabitants enjoy many of the same benefits as the married, which is not unlikely [21], the increase in cohabitation could contribute in explaining the less pronounced increase in the excess mortality of the never-married among the youngest in our analysis.

Another potential limitation is that we have restricted the analyses to 13 common cancer forms. If we included all other localizations into a 14^th ^category (i.e. not taking into account that some of these other cancer types are more aggressive than others and that the most aggressive types may occur more frequently in some marital status groups than others), very similar results were seen. This change in the analysis increased the sample size by one-third.

There were no obvious reasons to expect that the change over time in the relationship between marital status and cancer survival would differ across the 13 cancer types. The suggested mechanisms should be generally relevant, though perhaps with some differences in their relative importance, and earlier studies have not shown clear and interpretable differences across sites in the overall effect of marital status on cancer survival [51]. Therefore, we did not estimate models separately for each of the sites considered.

The corrected-survival approach that we have used may in principle not always give a good impression of how aggressive the disease is, because it is often difficult to identify a primary, underlying cause of death. An alternative would be the relative-survival approach, which is a comparison of all-cause mortality in cancer patients with that in individuals of the same age and sex in the "normal population" or even (as done in a few studies) those with similar marital status, education or other socio-demographic characteristics. This is, however, a more cumbersome procedure, and it has been shown that the results are almost identical with respect to marital status differentials [52]. Another alternative could be the observed-survival approach, where the focus is on *all-cause *mortality among cancer patients. We performed also these analyses, and very similar estimates resulted.

The patients' educational level is controlled for in the models, and this has some impact on the estimates. For the observations after 1980, it is the educational level during the preceding year that is included, while for earlier observations only the level in 1970 (which is up to ten years earlier) is available. In our study population only the few individuals in their low 30 s in this time period are likely to have experienced any changes in their educational level over the previous ten years, and the lack of continuous education data should thus not influence our results markedly.

This study has, however, several obvious strengths. The time-span covered is rather large, and the data include the entire Norwegian population. It is also important that we can control for tumor stage at diagnosis.

## Conclusions

Never-married cancer patients appear to have had increasingly poor survival prospects compared to the married over the last four decades. The picture is more blurred for the widowed, while there has been stability among the divorced. The adverse trend among the never-married is only seen for those diagnosed at ages above 70, which perhaps partly reflects an increasing proportion of cohabitants among the younger. There is no obvious explanation for the increasing disadvantage among the relatively old never-married. It could be due to a relatively poorer general health at the time of diagnosis in this group, either because of a more protective effect of partnership in a society that may have become less cohesive or because of a more positive selection into marriage. Alternatively, the trends observed in this study could be related to increasing differentials with respect to treatment. More specifically, the complexity of present cancer treatment regimens could be more difficult for the never-married to adhere to. They might thus be in need of closer follow-up from health care workers when it comes to treatment adherence.

All effects suggested here as potentially producing a change in the relationship between marital status and cancer survival should be broadly relevant, so it is reasonable to expect similar trends in many other countries. Should that be confirmed in later studies, an important next step is to learn more about the relative importance of the various mechanisms. One could for instance explore potential martial status differentials in type of surgery, use of radiation therapy or differences in chemotherapeutic drugs offered. Perhaps even more important is to investigate possible differentials in treatment compliance, e.g. the taking of medication, meeting to consultations, following the doctors' advices, and so on. Findings from such research may have important implications for future cancer treatment and care.

## Competing interests

The authors declare that they have no competing interests.

## Authors' contributions

HK planned and carried out the analyses, and wrote the paper. AS helped write the paper. Both authors read and approved the final manuscript.

## Pre-publication history

The pre-publication history for this paper can be accessed here:

http://www.biomedcentral.com/1471-2458/11/804/prepub
